# Pharmacokinetic Characterization of LW6, a Novel Hypoxia-Inducible Factor-1α (HIF-1α) Inhibitor in Mice

**DOI:** 10.3390/molecules26082226

**Published:** 2021-04-12

**Authors:** Ji-Yoon Lee, Kiho Lee, Kyeong Lee, Jong Soon Kang, Min Ju Kim, Dong Gu Yoo, Jung Ah Kim, Eun Jin Shin, Soo Jin Oh

**Affiliations:** 1 Convergence Medical Research Center, Asan Institute for Life Science, Asan Medical Center, Seoul 05505, Korea; lsuklee@hanmail.net; 2 Department of Pharmacy, College of Pharmacy, Korea University, Sejong 30019, Korea; kiholee@korea.ac.kr; 3 BK21 FOUR Team and Integrated Research Institute for Drug Development, College of Pharmacy, Dongguk University—Seoul, Goyang 10326, Korea; kaylee@dongguk.edu; 4 Laboratory Animal Resource Center, KRIBB, Chungbuk 28116, Korea; kanjon@kribb.re.kr (J.S.K.); redbijou@kribb.re.kr (M.J.K.); 5 Department of Medical Science, Asan Medical Institute of Convergence Science and Technology, Asan Medical Center, University of Ulsan College of Medicine, Seoul 05505, Korea; dbehdrn2992@naver.com (D.G.Y.); jung.kim0702@gmail.com (J.A.K.); aa0877@naver.com (E.J.S.)

**Keywords:** LW6, mice pharmacokinetics, liver microsomes, metabolism, Caco-2 cells

## Abstract

LW6, an (aryloxyacetylamino)benzoic acid derivative, was recently identified to be an inhibitor of hypoxia-inducible factor-1α (HIF-1α), which is an attractive target for cancer therapeutics. Although LW6 is known to act by inhibiting the accumulation of HIF-1α, pharmacokinetics needs to be evaluated to assess its potential as an anti-tumor agent. Here, we investigated the plasma pharmacokinetics and metabolism of LW6 in mice. LW6 exhibited a small volume of distribution (0.5 ± 0.1 L/kg), and a short terminal half-life (0.6 ± 0.1 h). Following intravenous or oral administration, LW6 was rapidly converted to its active metabolite, (4-adamantan-1-yl-phenoxy)acetic acid (APA). Although LW6 was rapidly absorbed, its oral bioavailability, estimated using *AUC_last_* values, was low (1.7 ± 1.8%). It was slowly degraded in mouse liver microsomes (*t*_1/2_ > 1 h) and serum (*t*_1/2_ > 6 h). About 54% or 44.8% of LW6 was available systemically as APA in the mouse after a single intravenous or oral administration, respectively. Thus, our results indicated the need to simultaneously consider the active metabolite as well as the parent compound for successful evaluation during lead optimization.

## 1. Introduction

The hypoxia-inducible factors (HIFs) are key transcriptional regulators that adaptively respond to changes in available oxygen in the cellular environment, specifically hypoxia [1]. HIF-1 is an oxygen-sensitive transcriptional activator and up-regulates transcription of the several genes that are involved in crucial aspects of cancer biology, including angiogenesis, cell survival, glycolysis, and invasion to promote survival in hypoxic conditions [2,3]. HIF-1 is a heterodimer that consists of an oxygen-sensitive HIF-1α subunit and a constitutively expressed HIF-1β subunit, known as the aryl hydrocarbon receptor nuclear translocator (ARNT) [4]. HIF-1 activity is dependent on the availability of the HIF-1α subunit, which is regulated by cellular oxygen levels. At normal oxygen levels, HIF-1α is degraded via the von Hippel-Lindau (VHL)-mediated ubiquitin-proteasomal pathway. However, during hypoxia, HIF-1α promptly accumulates in the cell. Stabilized HIF-1α translocates into the nucleus and makes a heterodimer with HIF-1β. This heterodimer binds to a specific DNA sequence within the hypoxia sensitive target gene promoter, called the hypoxia-response element (HRE), which leads to transcriptional activation of various genes involved in the tumor growth [5,6,7,8,9].

In animal models, inhibition of HIF-1α was shown to significantly reduce tumor growth, vascularization, and metastasis [10]. Moreover, there is clinical evidence to show that HIF-1α plays an important role in human cancer progression [11]. Therefore, HIF-1α represents an attractive molecular target for the development of novel cancer therapeutics [12,13]. As a result, a number of anticancer agents have been identified as HIF-1 activity inhibitors [14,15,16,17,18]. In animal models, all of these inhibitors have been shown to act by inhibiting the expression of HIF-1 target genes, reducing the HIF-1α protein levels, and impairing tumor growth.

In previous studies, it was reported that a novel compound, an (aryloxyacetylamino)benzoic acid derivative, LW6 inhibited the accumulation of HIF-1α [19] and decreased HIF-1α protein expression without affecting HIF-1β expression via regulation of VHL [20].

The objective of this study was to investigate the pharmacokinetics and metabolism of LW6 in male ICR mice to support its preclinical development as an antitumor agent. Our results indicate that the antitumor activity of LW6 in vivo may be attributed to both LW6 and the formation of APA, an active metabolite as LW6 was shown to be metabolized rapidly to APA in the body.

## 2. Results

### 2.1. Plasma Pharmacokinetics of LW6 and Its Metabolite, APA in Mice

#### 2.1.1. LW6

**Figure 1:**
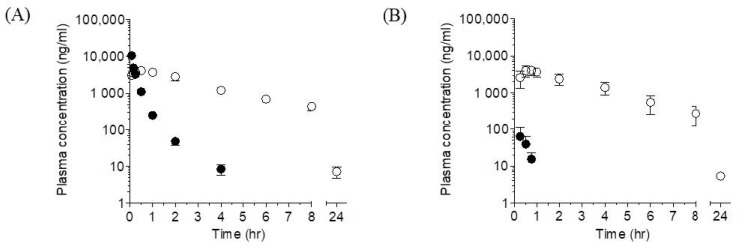
Plasma concentration-time profiles of LW6 and its metabolites following a single dose of LW6 in male ICR mice. A single dose of LW6 was given intravenously (**A**; 5 mg/kg; *n* = 4) or orally (**B**; 5 mg/kg; *n* = 5) and the plasma concentration of LW6 (●) and its metabolites, (4-adamantan-1-yl-phenoxy)acetic acid (APA) (○), was determined for 24 h post-dosing. Each point represents mean ± S.D. (*n* = 4~5).

In our preliminary study, APA was detected as a major metabolite of LW6 in mice. Therefore, male ICR mice were given a single intravenous (i.v., 5 mg/kg) or oral dose (p.o., 5 mg/kg) of LW6, and the plasma pharmacokinetics of both LW6 and its putative metabolite, APA was investigated simultaneously. The plasma concentration-time profiles of LW6 and APA, following the i.v. and p.o. administration of LW6, are shown in Figure 1. The calculated pharmacokinetic parameters of LW6 and APA obtained following a single dose of LW6 are presented in Table 1.

After i.v. administration of LW6 at a dose of 5 mg/kg, the plasma concentration declined rapidly in an apparent polyexponential fashion. The plasma level of LW6 was below the quantitation limit beyond 4 h (Figure 1A). An apparent terminal phase was defined in the plasma concentration-time curve of LW6 between 1 to 4 h post-administration with a *t*_1/2_ of 0.6 ± 0.1 h (Figure 1 and Table 1); the volume of distribution at steady state (*V_ss_*) was 0.5 ± 0.1 L/kg, close to the total body water volume (0.7 L/kg), indicating that LW6 was distributed outside the vasculature. The systemic clearance (*CL*) of LW6 was 1.7 ± 0.1 L/hr^/^kg (Table 1), lower than the hepatic blood flow of the mouse (Table 1). APA appeared rapidly in the plasma with its *t_max_* occurring at the early time point (i.e., 10 min or 15 min) in all animals, following 5 mg/kg i.v. dose (Figure 1A and Table 1). Mean plasma *t*_1/2_ of APA was 2.7 ± 0.2 h following the intravenous administration of LW6. *AUC*_0–*inf*_ of APA in the plasma was 6-fold greater than that of LW6 (Table 1).

The oral absorption of LW6 was rapid with its mean *t_max_* occurring at 0.3 ± 0.1 h, following 5 mg/kg oral dose in mice (Figure 1B and Table 1); its *t*_1/2_ and *AUC*_0–*inf*_ could not be determined following the oral administration due to the inability to define terminal elimination phase in the current study (Figure 1B). The plasma level of LW6 was below the quantitation limit beyond 1 h (Figure 1B). Plasma exposure of APA, as represented by *AUC*_0–*t*_, was 295-fold greater than that of LW6, following the oral administration of LW6 (Table 1). Similar to an intravenous dose, the mean plasma *t*_1/2_ of APA was 2.4 ± 0.6 h (Table 1). The mean oral bioavailability of LW6 was estimated using *AUC*_0–*t*_ values to be 1.7 ± 1.8 % (Table 1).

#### 2.1.2. APA

**Figure 2:**
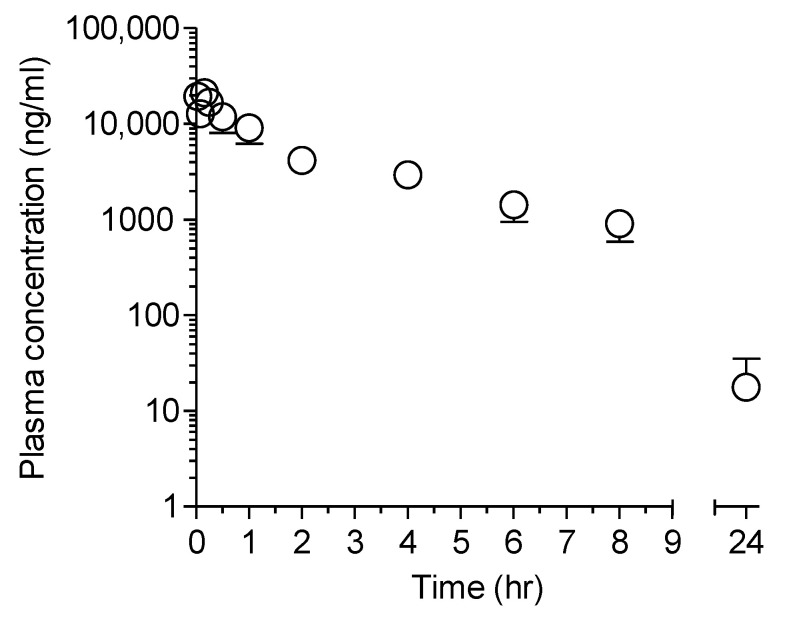
Plasma concentration-time profiles of APA following a single intravenous dose of APA in male ICR mice. A single dose of APA (5 mg/kg; *n* = 5) was given intravenously and the plasma concentration of APA (○) was determined for 24 h post-dosing. Each point represents mean ± S.D. (*n* = 5).

Plasma pharmacokinetics of APA was characterized in a separate experiment, following a single intravenous dose of APA in ICR mice. APA exhibited multi-compartmental disposition characteristics (Figure 2). The disposition kinetics of APA (Figure 2) was very similar to those obtained following the administration of LW6 (Figure 1A), with the *t*_1/2_ values almost identical in both cases (Table 1 and Table 2). The systemic clearance (*CL*) of APA was 0.1 ± 0.0 L/hr^/^kg (Table 2), which was much lower than the hepatic blood flow of the mouse. Its *V_ss_* value (0.4 ± 0.1 L/kg) was also relatively low (Table 2). 

### 2.2. Permeability of LW6 in Caco-2 Cells

To understand if intestinal permeation was the reason for the low oral bioavailability of LW6, its permeability was investigated using Caco-2 cells, derived from colon adenocarcinoma.

The *P_app_* value of LW6 in the apical-to-basolateral direction was 2.1 × 10^−6^ cm/s, that was between those of metoprolol, the high permeability reference, and ranitidine and atenolol, the low permeability references (Table 3), suggesting that it is likely to be moderately permeable in the intestine. APA was not detected either at the donor or the receiver sides during the transport experiment (data not shown). 

### 2.3. Metabolic Stability of LW6 in Mouse Liver Microsomes and Serum

**Figure 3:**
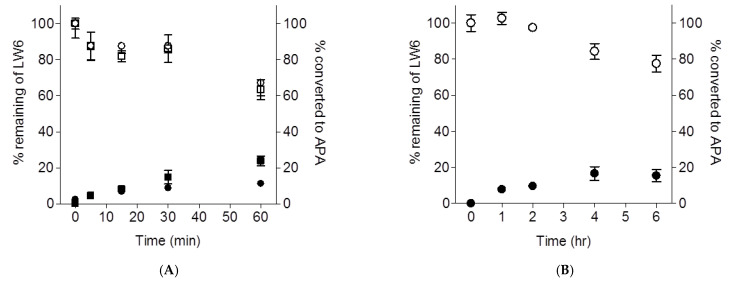
Time course of LW6 depletion and the formation of its hydrolyzed metabolite, APA, following incubation of LW6 in mouse liver microsomes (**A**) and serum (**B**). LW6 (1 µM) was incubated with 0.5 mg/mL of pooled mouse liver microsomes in the absence (square) or presence (circle) of NADPH and LW6 (5 µM) was incubated in mouse serum. Following incubation, the concentration of LW6 (opened ○ or □) and APA (closed ● or ■) was determined at selected time points by LC-MS/MS (see Figure 3). Each point represents the mean ± S.D. of triplicate determinations.

**Figure 4:**
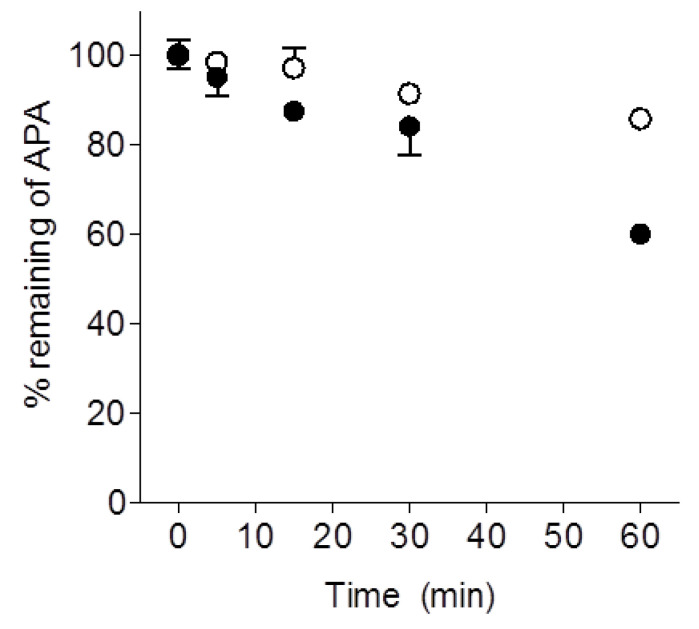
Time course of APA depletion following incubation of APA in mouse liver microsomes. APA (1 µM) was incubated with 0.5 mg/mL of pooled mouse liver microsomes in the absence (○) or presence (●) of NADPH. Following incubation, the concentration of APA was determined at selected time points by LC-MS/MS (see Figure 3). Each point represents the mean ± S.D. of triplicate determinations.

LW6 (1 µM) was incubated with pooled mouse liver microsomes (0.5 mg/mL) in the absence or presence of NADPH (1 mM) to determine its conversion to APA. LW6 was degraded slowly in the absence or presence of NADPH, with 63% or 65% remaining after 60 min microsomal incubations, respectively (Figure 3A). LW6 was converted slowly to APA (*t*_1/2_ > 60 min) in a quantitative manner in liver microsomes (Figure 3A). APA, following its formation, gradually disappeared from the microsomal incubation media only in the presence of NAPDH (Figure 3A). To determine whether APA was metabolized by cytochrome P450 (CYP450), APA (1 µM) also was incubated with pooled male mouse liver microsomes (0.5 mg/mL) in the absence or presence of NADPH (1 mM). As shown in Figure 4, APA was progressively decreased when incubated with mouse liver microsomes in the presence of NADPH. These results suggested that LW6 was metabolized to APA, which was further metabolized by CYP450. In the case of mouse serum, LW6 was also converted slowly to APA (Figure 3B).

## 3. Discussion

The present study demonstrates that LW6 was converted rapidly to its active metabolite APA in mice. Consistent with this result, the plasma concentration-time profile and the terminal elimination half-life for formed APA following LW6 administration remained fairly similar whether LW6 or APA itself was administered via the intravenous route. These results indicated that the plasma pharmacokinetics of APA following the administration of LW6 was determined predominantly by its elimination kinetics rather than its formation kinetics. It is apparent that LW6 was also rapidly converted to APA following an oral dose of LW6, as evidenced by the observation that the *t_max_* of APA occurred early time point. The *t_max_* values of APA were very close following an intravenous or oral dose of LW6: 0.6 ± 0.3 h or 0.7 ± 0.1 h for an intravenous or oral dose, respectively (Table 1). These data suggested that the plasma pharmacokinetics of APA following an oral dose of LW6 was not affected by the absorption process of LW6. The Caco-2 permeability study showed that LW6 was moderately permeable across Caco-2 cell monolayers (Table 3). 

The elimination kinetics of APA as represented by the terminal elimination half-life, which was similar to its half-life after intravenous (*t*_1/2_ = 2.7 h) or oral (*t*_1/2_ = 2.4 h) dose of LW6. The amount of APA available in the systemic circulation well exceeded that of LW6 relative to LW6 dose (Table 1 and Table 4).

The plasma exposure of LW6 was significantly decreased to about 60-fold when it was administered orally. Thus, the bioavailability of LW6 was very low (1.7 ± 1.8 %). Nevertheless, the plasma exposure of APA was very similar when LW6 was given both orally and intravenously in the mouse: the *C_max_* values were 4210.0 ± 823.1 ng/mL or 4236.0 ± 1262.0 ng/mL for an intravenous or oral dose, respectively (Table 1). The *AUC*_0–*inf*_ values were 17,759.9 ± 3194.1 ng/hr/mL or 14,744.8 ± 5517.4 ng/hr/mL for intravenous or oral dose, respectively (Table 1). The ratio of plasma exposure of APA relative to LW6 increased significantly when LW6 was given orally vs. intravenously in the mouse: the ratios were 6 (intravenous) vs. 295 (oral) for *AUC*_0–*t*_. These results indicated that APA was not only formed but also made available systemically during gastrointestinal absorption and first-pass metabolism following the oral dose of LW6.

LW6 was shown to be converted to APA in mouse serum and liver microsomes in vitro, consistent with the in vivo results described above, although the kinetic of APA formation in vitro is slower than that of in vivo. In the present study, APA were formed and increased during the incubation of LW6 without NADPH in the mouse liver microsomes. This metabolic transformation was likely due to hydrolase activities, as LW6 was shown to be stable in phosphate buffer at physiological pH (data not shown). Whereas, the concentration of APA formed from LW6 was decreased during the microsomal incubation in the presence of NAPDH. APA contains an adamantane skeleton, which is known to be hydroxylated by cytochrome P450 [21,22]. Although additional studies are required to characterize the metabolism of LW6 further, it can be postulated based on the biotransformation of adamantane that hydroxylation of LW6 and formed APA by NADPH-dependent metabolizing enzymes, cytochrome P450s could have caused the loss of formed APA in microsomal incubation with NADPH.

About 54% or 44.8 % of administered LW6 was shown to be available systemically as APA in mice following a single intravenous or oral dose, respectively (Table 4). The remaining dose of LW6 remained largely unaccounted for at the moment; however, it is probably eliminated by other pathways such as oxidation, glucuronidation, another phase II metabolism, and excretion. It is also possible that the majority of APA was eliminated at the site of formation before reaching the systemic circulation. 

Although LW6 exhibits inadequate pharmacokinetic characteristics for an optimal drug candidate, i.e., low systemic exposure, short *t*_1/2_*,* and low bioavailability, LW6 is currently under preclinical development based on its antitumor activity as well as low toxicity. The apparent disconnect between pharmacokinetics and pharmacodynamics warrants further investigation.

Preclinical pharmacokinetics studies play a critical role in leading identification and optimization in the early drug discovery process. The present study is the first preclinical pharmacokinetic evaluation of this lead compound of novel HIF-1α inhibitor series. Results from the HCT116 xenograft model study showed that APA significantly inhibited the tumor growth up to 43.3%, relative to the control group (data not shown). These results suggested that LW6 as an anticancer drug is highly likely to work as its active metabolite APA in the body. Therefore, it would be essential to simultaneously consider the active metabolite as well as the parent compound for successful evaluation during this lead optimization.

## 4. Materials and Methods

### 4.1. Chemicals and Reagents

**Figure 5:**
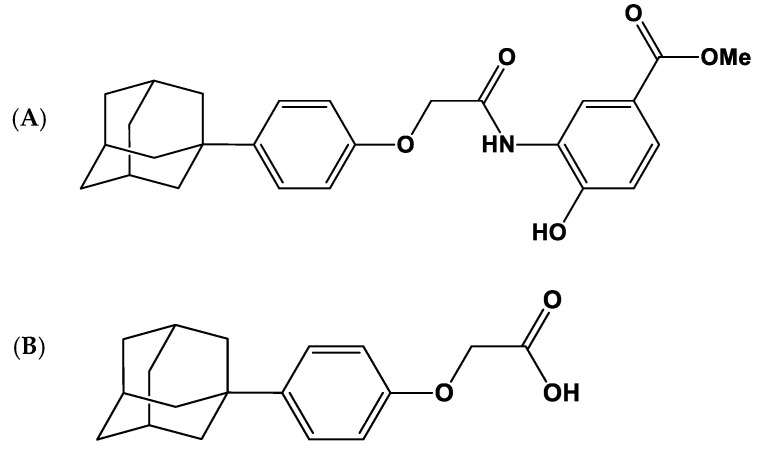
Structures of LW6 (**A**) and its metabolite, 2-(4-((3R, 5R, 7R)-adamantan-1-yl phenoxy)acetic acid (APA) (**B**).

LW6 (>96.04% purity) and 2-(4-((3*R*, 5*R*, 7*R*)-adamantan-1-yl phenoxy)acetic acid (APA, >99.99% purity) were provided by Dr. Kyeong Lee at College of Pharmacy, Dongguk University (Seoul, Republic of Korea) (Figure 5). 

β-Nicotinamide adenine dinucleotide phosphate reduced (β-NADPH), dimethylacetamide, Cremophor EL, 2-hydroxypropyl β-cyclodextrin, metoprolol, atenolol, ranitidine, and 4-methylumbelliferone were purchased from Sigma-Aldrich Co. (St. Louis, MO, USA). Sarstedt’s Microvette^®^ capillary tubes were purchased from Sarstedt (Nümbrecht, Germany). Minimum essential medium (MEM), non-essential amino acid (NEAA) solution, fetal bovine serum (FBS), 4-(2-hydroxyethyl)-1-piperazineethanesulfonic acid (HEPES) solution (1 M), 0.25% trypsin-EDTA, penicillin-streptomycin solution, phosphate-buffered saline (PBS), and Hanks’ balanced salt solution (HBSS) were purchased from Invitrogen (Carlsbad, CA, USA). Transwells^TM^ (12 mm i.d., 3.0 μm pore size, polycarbonate membrane, 12-well) were purchased from Costar (Cambridge, MA, USA). Pooled mouse liver microsomes (28596, pool of 585 donors) were purchased from BD Gentest (Woburn, MA, USA). Pooled mouse serum was freshly prepared on the day of the experiment from ten male ICR mice (9 weeks old). Acetonitrile (HPLC grade) was purchased from Fisher Scientific Co. (Pittsburgh, PA, USA) and all other chemicals were of the highest quality available.

### 4.2. In Vivo Pharmacokinetic Studies

The animal experiments conducted in this study were approved by the Institutional Animal Care and Use Committee of the Korea Research Institute of Bioscience and Biotechnology (Chung-buk, Korea; KRIBB-AEC-15005, 9 January 2015) and performed in compliance with the National Institutes of Health Guidelines for the care and use of laboratory animals and Korean national laws for ethical conduct in the care and use of animals. Specific pathogen-free (SPF) male ICR mice (8 weeks old) were purchased from Koatech Co. (Pyeongtaek, Kyonggi, Republic of Korea) and maintained in an SPF environment at a temperature of 22 ± 2 °C with a 12-h light/dark cycle and a relative humidity of 50 ± 10%. Food and water were given ad libitum. Animals were allowed to acclimatize to the testing facility for a week before being used in the study. Animal group for oral administration was fasted overnight before the study and fed again 4 h after dosing. Mice were given a single dose of test substance intravenously (i.v.) via tail vein injection or orally (p.o.) by a disposable syringe with an oral zonde. While LW6 was administered i.v. (5 mg/kg) or p.o. (5 mg/kg), APA was administered only i.v. (5 mg/kg). The solutions were prepared in dimethylacetamide/Cremophor EL/20% *v*/*v* 2-hydroxypropyl β-cyclodextrin in de-ionized water (1/1/3 *v*/*v*; i.v. or p.o. for LW6) or ethanol/20% *v*/*v* 2-hydroxypropyl β-cyclodextrin in de-ionized water (1/9 *v*/*v*; i.v. for APA) and administered at a dose volume of 5 mL/kg for both i.v. and p.o. Blood samples were collected via saphenous vein without the use of anesthesia as described in the earlier methodology study [23] at the time points including 0, 0.25, 0.5, 0.75, 1, 2, 4, 6, 8, and 24 h after oral administration or 0, 0.083, 0.167, 0.25, 0.5, 1, 2, 4, 6, 8, and 24 h after intravenous administration. These samples were collected into Microvette^®^ capillary tubes at predetermined time points. The blood samples were centrifuged under refrigeration at 12,000× *g* for 3 min and then kept frozen at −20 °C until analysis.

#### 4.2.1. Sample Analysis

Plasma samples were prepared for analysis by the protein precipitation method. An aliquot of 15 µL from each plasma sample was transferred to a PCR tube (Axygen, Union City, CA, USA). Four volumes of acetonitrile containing the analytical internal standard (IS) 4-methylumbelliferone were added and the resulting mixture was vortexed for 10 min on a Multi-Tube vortexer (VWR International, West Chester, PA, USA), and sonicated for 30 min at room temperature. The tube was centrifuged at 12,000× *g* for 10 min and the supernatant was analyzed for the test substance. Sample analysis was performed by 3200 Q TRAP LC-MS/MS system (Applied Biosystems, Concord, ON, Canada) in a negative MRM mode. The analytical methods are described below.

#### 4.2.2. Data Analysis

Pharmacokinetic parameters were calculated by noncompartmental analysis of the plasma concentration-time profiles using Kinetica^TM^ 4.4.1 (Thermo Fisher Scientific, Inc., Woburn, MA, USA). The areas under the plasma concentration–time curves (*AUC*) were calculated by the linear-trapezoidal method. Total body clearance (*CL*) was calculated as follows: *CL* = *Dose/AUC*_0–*inf*_. Terminal elimination half-life (*t*_1/2_) was calculated by the following equation: *t*_1/2_ = 0.693/*λz*, where *λz* is the terminal disposition rate constant. Volume of distribution at steady state (*V_ss_*) was calculated as follows: *V_ss_ = CL* × *AUMC*_0–*inf*_*/AUC*_0–*inf*_, where *AUMC*_0–*inf*_ is the area under the first moment of the plasma concentration-time curve extrapolated to infinity. Oral bioavailability (*F*) was calculated using the following equation:

(1) $$F=\frac{\left(AUC_{po}/Dose_{po}\right)}{\left(AUC_{iv}/Dose_{iv}\right)}\times 100$$

Fraction of dose converted to a systemically available metabolite was calculated by the following equation, where *D* is the dose of the parent, *AUC*(*m*) is the area under the curve of metabolite, and *CL*(*m*) is the systemic clearance of metabolite:

(2) $$\%\;\mathrm{of}\;\mathrm{dose}\;=\;\frac{AUC(m)\;\times CL(m)}{D}\times \frac{\mathrm{parent}\;\mathrm{mol}.\;\mathrm{wt}.}{\mathrm{metabolite}\;\mathrm{mol}.\;\mathrm{wt}.}\;\times 100$$

### 4.3. Permeability Assay in Caco-2 Cell Monolayers 

Caco-2 cells were obtained from ATCC (Rockville, MD) at passage 18, and cultured as described previously [24] with minor modifications. Briefly, Caco-2 cells were cultured at 37 °C in MEM, supplemented with 10% FBS, 1% NEAA, 100 U/mL penicillin, and 100 μg/mL streptomycin in an atmosphere of 5% CO_2_ and 90% relative humidity. The cells were passaged every 4–6 days using trypsin-EDTA at a split ratio of 1:20 at about 90% confluency. Caco-2 cells (passage 22~25) were seeded at a density of 60,000 cells/cm^2^ on polycarbonate membranes of Transwells^TM^ (12 mm i.d., 3.0 µm pore size). The medium was changed the day after seeding and every other day thereafter with an apical (AP) volume of 0.5 mL and a basolateral (BL) volume of 1.5 mL. The cell monolayers were used 20~25 days post-seeding. Transepithelial electrical resistance (TEER) was measured using EVOM Epithelial Tissue Voltohmmeter (World Precision Instruments, Sarasota, FL, USA) and STX2 “chopstick” electrode (World Precision Instruments, Sarasota, FL). TEER values before and after application of LW6 were 738.0 ± 14.0 and 636.0 ± 58.1, respectively. The configuration of the cell layer stayed relatively constant during the experiment. Monolayers having TEER values above 400 Ω cm^2^ were used in studies. Cell monolayer integrity was checked also by determining the AP-to-BL permeability of low (ranitidine and atenolol) and high (metoprolol) permeability references (FDA Guidance for Industry). Typical apparent permeability coefficient (*P_app_*) values were <2, <1, and >25 × 10^−6^ cm/s for ranitidine, atenolol, and metoprolol, respectively.

#### 4.3.1. Transport Studies

Transport experiments were performed as described previously [24] with some modifications. All studies were conducted with HBSS supplemented with 10 mM HEPES (pH 7.4). Cell monolayers were preincubated for 30 min at 37 °C with the study buffer in both the apical (AP) and basolateral (BL) sides, and TEER value was measured. The inserts were then transferred to a 12-well cell culture plate (BD, Franklin Lakes, NJ, USA) containing 1.5 mL of prewarmed study buffer (pH 7.4, 1% *v*/*v* DMSO) in each well. Transport experiments were initiated by replacing the AP side of the cell monolayers with 0.5 mL of test substance solution (5 µM) in the study buffer (pH 7.4, 1% *v*/*v* DMSO). The cell monolayers were then incubated at 37 °C on a rocker operated at 30 rpm. Experiments were terminated at 120 min by removing the inserts. Aliquots (100 µL) of both the AP and BL solutions were transferred to a 96-well plate followed by dilution with an equal volume of acetonitrile containing the internal standard for sample analysis. The analytical methods are described below. TEER value was measured thereafter to determine if the integrity of the cell monolayers were maintained during the experiments.

#### 4.3.2. Data Analysis

*P_app_* (cm/s) value was calculated using the following equation:

(3) $$P_{app}=\frac{1}{AC_{0}}\frac{dQ}{dt}$$

where *dQ/dt* is the rate of appearance (nmol/s) in the receiver side determined experimentally by measuring the amount of the test substance transported as a function of time; *A* is the surface area of the membrane (cm^2^); *C*_0_ is the initial concentration (nmol/mL) of the test substance in the donor side. 

### 4.4. In Vitro Incubations

#### 4.4.1. Mouse Serum

All incubations were conducted in triplicate at 37 °C in eight-well tube strips placed in an 8 × 12 rack (VWR, Emeryville, CA, USA) with 50 µL of mouse serum in each tube. LW6 (1 mM) dissolved in acetonitrile was added to a final concentration of 5 µM (1% acetonitrile) to initiate the study. The reaction was terminated at 0, 1, 2, 4, and 6 h by adding 150 µL of ice-cold acetonitrile containing the internal standard. The eight-well cluster tube plate was centrifuged at 910× *g* for 10 min and the supernatant was analyzed by the LC-ESI/MS/MS method described below for quantitation of LW6 and APA.

#### 4.4.2. Mouse Liver Microsomes

All incubations were conducted in triplicate at 37 °C in eight-well tube strips placed in an 8 × 12 rack (VWR, Emeryville, CA). Incubation mixtures consisted of mouse liver microsomes (0.5 mg protein/mL), NADPH (1 mM), and LW6 (1 µM) or APA (1 µM) in 0.1 M potassium phosphate buffer (pH 7.4). The final incubation volume was 160 µL and the final acetonitrile content was 1%. Samples were preincubated for 5 min at 37 °C before adding LW6 or APA to initiate the reaction. The reaction was terminated at 0, 5, 15, 30, and 60 min by the addition of 320 µL of ice-cold acetonitrile containing the internal standard. Incubation mixtures were then centrifuged at 910× *g* for 10 min and the supernatant was analyzed by the LC-ESI/MS/MS method described below for quantitation of LW6 and APA.

### 4.5. Bioanalytical Methods

**Figure 6:**
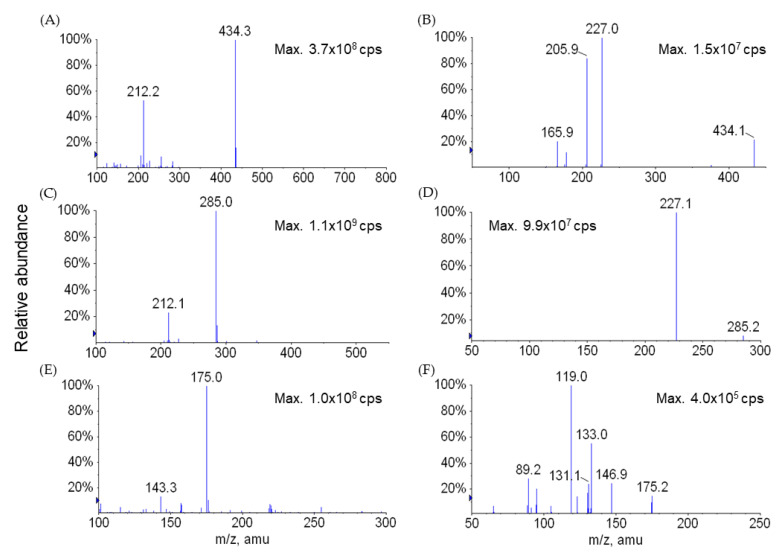
ESI negative Q1MS (**A**,**C**,**E**) and MS/MS product ion (**B**,**D**,**F**) spectra for LW6 (**A**,**B**), APA (**C**,**D**), and the internal standard (**E**,**F**).

**Figure 7:**
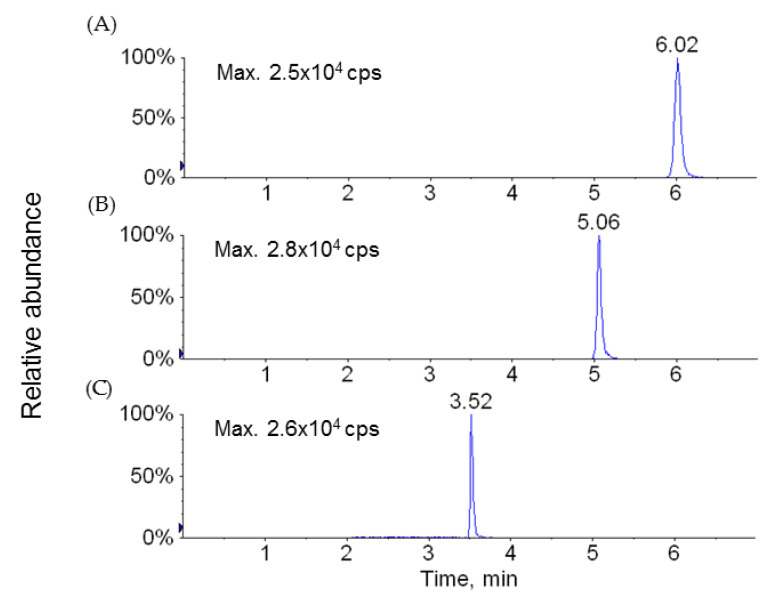
Representative MRM chromatograms acquired from LC-MS/MS analysis of LW6 (**A**, *m*/*z* 434→227), APA (**B**, *m*/*z* 285→227), and the internal standard (**C**, *m*/*z* 175→133).

The LC-MS/MS system consisted of an Agilent 1100 series HPLC system (Agilent Technologies, Wilmington, DE, USA) and a 3200 QTRAP LC-MS/MS system (Applied Biosystems, Foster City, CA, USA) equipped with a Turbo V^TM^ ion spray source operated in the negative ion mode. Sample injection volume was 10 µL and the separation was performed on an Atlantis dC18 column (50 × 2.1 mm i.d., 3 µm, Waters, Milford, MA, USA) with a SecurityGuard^TM^ C18 guard column (2.0 × 4.0 mm i.d., Phenomenex, Torrance, CA, USA) maintained at room temperature. The column was preequilibrated in 80% *v*/*v* solvent A (deionized water containing 0.5% *v*/*v* acetic acid)/20% *v*/*v* solvent B (acetonitrile containing 0.5% *v*/*v* acetic acid) at a flow rate of 0.4 mL/min. A linear gradient of the two solvents was used: start at 80% A and hold for 1 min, ramp to 20% A to 1.5 min and hold until 7 min. The flow rate was set at 0.4 mL/min throughout the gradient. The retention times of LW6, APA, and the internal standard were 6.0, 5.1, and 3.5 min, respectively. The ESI source was operated at −4500 V and 600 °C. The typical ion source parameters were as follows: declustering potential (DP): −68 (LW6), −45 (APA), and −45 eV (IS); collision energy (CE): −40 (LW6), −30 (APA), and −28 eV (IS); entrance potential (EP): −7 (LW6), −8 (APA), and −12 V (IS); collision cell exit potential (CXP): −18 (LW6), 0 (APA), and 0 (IS). Nebulizer gas (NEB), curtain gas (CUR), and collision gas (CAD) were set to 50, 30 psi, and medium, respectively. Nitrogen gas was used for NEB, CUR, and CAD. Quadrupoles Q1 and Q3 were set on unit resolution. The samples were analyzed via multiple reaction monitoring (MRM). The monitoring ions were set as *m/z* 434→227 for LW6, *m/z* 285→227 for APA, and *m/z* 175→133 for the IS. The scan dwell time was set at 0.1 sec for every channel. Acquisition and analysis of data were performed with Analyst^TM^ software (version 1.4.2, Applied Biosystems, Foster City, CA). Representative MS spectra and chromatogram from this LC-MS/MS analysis are shown in Figure 6 and Figure 7, respectively. 

Calibration standards (3.9–4000 ng/mL, 4.9–5000 nM, 3.9–2000 nM, and 4.9–5000 nM for the pharmacokinetic studies, the Caco-2 transport studies, microsomal stability study, and serum stability study, respectively) were prepared in blank matrices pretreated with ice-cold acetonitrile containing the internal standard, 4-MUF; the pretreatment of blank matrices was necessary due to the instability of the analytes in the matrices. Calibration curves constructed using linear least-squares regression were linear over the concentration range of the standards used (*r*^2^ > 0.997). Relative standard deviation (RSD) of the measured concentrations was used to assess the precision. Comparison of the mean measured concentration versus the corresponding nominal concentration was used to assess the accuracy. Both the accuracy (80–120%) and precision (RSD < 20%) of the assay were acceptable.

## Figures and Tables

**Table 1:** Pharmacokinetic parameters were obtained following a single dose of LW6 in mice.

Parameter	i.v.		p.o.	
	LW6	APA	LW6	APA
*Dose* (mg/kg)	5 (*n* = 4)		5 (*n* = 5)	
*t_max_* (hr)	NA	0.6 ± 0.3	0.3 ± 0.1	0.7 ± 0.1
*C_max_* (ng/mL)	NA	4210.0 ± 823.1	65.5 ± 47.3	4236.0 ± 1262.0
*AUC*_0*–t*_ (ng/hr/mL)	2949.3 ± 218.4	17,731.4 ± 3190.5	49.5 ± 52.5	14,615.3 ± 5507.8
*AUC*_0–*inf*_ (ng/hr/mL)	2957.1 ± 215.4	17,759.9 ± 3194.1	NC	14,744.8 ± 5517.4
*CL* (L/hr/kg)	1.7 ± 0.1	NA	NA	NA
*V_ss_* (L/kg)	0.5 ± 0.1	NA	NA	NA
*V_z_* (Lkg)	1.6 ± 0.3	NA	NA	NA
*t*_1/2_ (hr)	0.6 ± 0.1	2.7 ± 0.2	NC	2.4 ± 0.6
* MRT * (hr)	0.3 ± 0.0	3.6 ± 0.2	NC	3.2 ± 0.3
* F * (%)	NA	NA	1.7 ± 1.8	NA

Pharmacokinetic parameters were calculated by noncompartmental analysis of the plasma concentration-time curves obtained following a single dose of LW6 (5 mg/kg for both i.v. and p.o., respectively) in male ICR mice. Values are mean ± S.D. (*n* = 4~5). NA: not applicable, NC: not calculated, F was calculated using *AUC*_0*–t*._

**Table 2:** Pharmacokinetic parameters obtained following a single intravenous dose of APA in mice.

Parameter	APA Dosed
*Dose* (mg/kg)	5
*AUC*_0–*t*_ (ng/hr/mL)	37,703.8 ± 10,162.9
*AUC*_0–*inf*_ (ng/hr/mL)	39,624.7 ± 9502.30
*CL* (L/hr/kg)	0.1 ± 0.0
*V_ss_* (L/kg)	0.4 ± 0.1
*V_z_* (L/kg)	0.5 ± 0.2
*t*_1/2_ (hr)	2.4 ± 0.4

Pharmacokinetic parameters were calculated by noncompartmental analysis of the plasma concentration-time curves obtained following a single dose of APA in male ICR mice. Values are mean ± S.D. (*n* = 5).

**Table 3:** Permeability of LW6 in Caco-2 monolayer.

Compound	*P_app_* × 10^−6^ (cm/s)
LW6	2.1 ± 0.4
Metoprolol	35.3 ± 0.6
Atenolol	0.4 ± 0.1
Ranitidine	0.8 ± 0.0

*P_app_* values were determined in the AP-to-BL direction. Values are mean ± S.D. (*n* = 3).

**Table 4:** Estimated fraction of dose available systemically as APA following a single administration of LW6 in mice.

Produced Metabolite	Dose	AUC(m)_0–*inf*_	CL(m)	Fraction of Dose
	(mg/kg)	(ng/hr/mL)	(L/hr/kg)	(%)
LW6 dosed				
*Intravenous*	5			
APA	NA	17,759.9 ± 3194.1	0.1 ± 0.0	54.0 ± 9.7
*Oral*	5			
APA	NA	14,744.8 ± 5517.4	0.1 ± 0.0	44.8 ± 16.8

The fraction of dose was calculated using the equation described in Section 4. Other data were taken from Table 1 and Table 2. Values are mean ± S.D. (*n* = 4~5). NA: not applicable.

## Data Availability

Data is contained within the article.

## Sample Availability

Samples of the compounds presented in the paper are available on request from the corresponding author.
